# Frailty in older adults admitted to hospital: outcomes from the Western Sydney Clinical Frailty Registry

**DOI:** 10.1186/s12877-025-05715-0

**Published:** 2025-02-04

**Authors:** Julee McDonagh, Richard I Lindley, Karen Byth, Reejamol John, Caleb Ferguson

**Affiliations:** 1 School of Nursing, Faculty of Science, Medicine & Health, University of Wollongong, Wollongong, NSW 2522 Australia; 2 Centre for Chronic and Complex Care Research, Blacktown Hospital, Western Sydney Local Health District, Blacktown, NSW 2148 Australia; 3 Westmead Applied Research Centre, University of Sydney, Westmead, NSW 2145 Australia; 4 Western Sydney Local Health District, Research and Education Network, Westmead, NSW 2145 Australia

**Keywords:** Frailty, Clinical Frailty Scale, Older adults, Rehospitalisation, Mortality

## Abstract

**Objectives:**

To examine baseline frailty and its association with rehospitalisation and mortality within 12 months among older adults enrolled in the Western Sydney Clinical Frailty Registry.

**Design:**

Prospective observational cohort study.

**Setting and participants:**

592 adults admitted to an acute geriatric medicine service in NSW, Australia, were included in this study.

**Methods:**

The Western Sydney Clinical Frailty Registry is a study of adults admitted to acute geriatric wards in a 570-bed two-site district general hospital in Western Sydney, NSW, Australia. Recruitment began in April 2020 and is ongoing. Each participant is recruited while an inpatient and followed up for 12 months, including baseline visits and three-, six- and 12-month follow-ups via telephone interviews. The primary outcome of this study was rehospitalisation and/or mortality at 12 months.

**Results:**

Median age 82 years; half the cohort were classified as mild-moderately frail, and 21% were classified as severely frail. A total of 134 participants died (22.6%) within the 12-month follow-up period. Increased cumulative incidence of first rehospitalisation and/or death during the first 12 months post-discharge was significantly associated with higher modified Charlson comorbidity (p < 0.001) and Clinical Frailty Scale (CFS) scores (p < 0.001). Compared to the ‘non-frail’ group (CFS 1–4), those who were severely frail (CFS 7–9) had an 85% increased risk of rehospitalisation and/or death (95% CI 1.36–2.52), and those who were mild-moderately frail (CFS 5–6) had a 52% increased risk after adjusting for effects of the other variables (95% CI 1.18–1.94).

**Conclusions:**

Frailty is very common in older adults admitted to acute geriatric services. Assessing frailty using the CFS is feasible and is independently predictive of rehospitalisation and mortality. Our findings suggest that integrating frailty assessment into clinical practice goes beyond simple risk stratification, offering valuable insights for tailored clinical management strategies.

## Introduction

Frailty is a common yet complex syndrome of vulnerability and physiological decline that can occur in some individuals alongside ageing or in the context of chronic disease and multimorbidity [1, 2]. Frailty as a concept has captured the attention of researchers, healthcare professionals and policymakers worldwide due to its significant impacts on the well-being of aging populations [3]. If a person is frail, they may experience adverse events and be at a greater risk of physical and cognitive decline, reduced mobility, falls, depression, frequent and prolonged hospital visits, and death [4, 5]. Frail older adults who present to hospital have complex care needs, such as multiple chronic conditions and increased risk of falls, and this can have significant impacts on their long-term outcomes [6]. Currently, public hospital clinicians working in acute geriatric settings throughout Australia do not routinely get feedback on their inpatients’ short- or longer-term outcomes, with only a proportion of their patients followed up in hospital services such as the outpatient clinic or outreach team. The Western Sydney Clinical Frailty Registry was established to obtain a baseline clinical profile of patients with frailty admitted to Rehabilitation and Aged Care services, as well as cross-sectional data regarding inpatient management and longitudinal patient outcome data. The Western Sydney Frailty Registry is the first established Australian frailty registry and currently the only frailty registry listed on the Australian Registry of Clinical Quality Registries [7].

Routine frailty screening is recommended for older adults [8]. However, several barriers, such as confusion regarding which frailty instrument to use and lack of time and resources, have limited the implementation of routine frailty screening in clinical practice [9]. Therefore, further research regarding frailty and its implications for older adults in acute care settings is needed to advanced practice and provide further evidence of the utility and prognostic value of incorporating frailty assessment into routine clinical practice.

Objective.

To examine baseline frailty and its association with rehospitalisation and mortality within 12 months among older adults enrolled in the Western Sydney Clinical Frailty Registry.

## Methods

### Design

The study methods have been previously reported [10]. The Western Sydney Clinical Frailty Registry is a prospective observational cohort study of adults admitted to the Rehabilitation and Aged Care Department at Blacktown and Mount Druitt Hospitals (a 570-bed, two-site district general hospital in Western Sydney, New South Wales, Australia). Recruitment began in April 2020 and is ongoing. Each participant is recruited while an inpatient at the study sites and followed up for 12 months, including baseline visits and three-, six- and 12-month follow-ups via telephone interviews; there is opportunity for future data linkage studies. At the baseline visit, data was collected through physical assessments, questionnaires, and extraction of routinely collected clinical data from Electronic Medical Records (eMR)–Cerner Millennium. Sociodemographic information, medical history, last available blood test results, current medication, physical assessment, cognitive and physical frailty and functional assessment data and details of discharge status were recorded.

Follow-up data were collected via telephone interview. If a participant could not be contacted after several attempts, information regarding death and hospitalisation was obtained from the participant’s medical record or public death registry data, as per study protocol.

### Setting and participants

A convenience sample of participants enrolled into the Western Sydney Clinical Frailty Registry with their 12-month follow-up completed. Patients admitted under Rehabilitation and Aged Care Services at Blacktown and Mount Druitt hospitals were eligible for inclusion (aged 65 years or older or otherwise admitted under the care of the service). People who could not consent in English or did not have a family member or caregiver to provide proxy consent, those under public guardianship or overseas visitors, and those for whom follow-up was not possible were excluded. Witnessed informed consent was obtained before the enrolment of all participants.

### Study measures

The primary outcome of this study was a composite endpoint: time to first rehospitalisation (including emergency department visits) and/or mortality at 12 months. A composite endpoint was chosen to avoid problems with competing risks. By combining rehospitalisation and mortality into a single outcome, competing events are less likely to preclude the occurrence of the primary event, thus enabling a more comprehensive analysis of both outcomes. Various sociodemographic and clinical data were collected at baseline including age, sex, past medical history and comorbidities (using the Modified Charlson Comorbidity Index [11]). Number of medications prescribed (polypharmacy was defined as > 5 medications prescribed [12]) and physical/functional status (using the Australian-modified Karnofsky Performance scale [AKPS]: a measure of the patient’s overall performance status scored between 10 and 100). An AKPS score of 100 signifies normal physical abilities with no evidence of disease. Decreasing numbers indicate a reduced performance status [13]). Cognitive function and delirium (using the routinely collected 4 A’s test comprising four items: Item 1 assesses level of alertness, Item 2 the Abbreviated Mental Test—4, Item 3 attention testing with months backwards, Item 4 assesses acute change or fluctuation in mental status -a score of 4 or more suggests delirium, a score of 1–3 suggests cognitive impairment [14]). An assessment of ‘pre-morbid’ (pre-hospital status) frailty was completed for all participants using the nine-item Clinical Frailty Scale (CFS). The CFS is an inclusive numeric scale first developed by Rockwood and colleagues in 2005 and revised in 2008 to summarise the overall level of frailty [15]. An individual is assessed as a score from 1 to 9, the higher the score, the greater the risk. This scale is not a questionnaire but requires clinical judgment based on the screening criteria provided. The screening criteria includes items that can be easily observed without needing specialist training, including, balance, mobility, walking aids, and ability to undertake activities of daily living (eating, dressing etc) [16]. The CFS was selected as it is quick and easy to complete, in a time-restricted setting, and has demonstrated strong utility and validity in acute aged care settings [17–19].

All data were entered into and stored in a purpose-built REDcap electronic database [20] hosted securely by the New South Wales Health Office of Health and Medical Research, Australia.

### Statistical analysis

All analyses were undertaken under the guidance of a senior biostatistician. IBM SPSS Statistics version 29 software (IBM, Amarok, NY) was used to analyse the data. To demonstrate the differences in baseline characteristics between the three frailty groups (non-frail, mild-moderate frail and severely frail), the sociodemographic and clinical data collected at baseline were tabulated by frailty group (CFS 1–4, 5–6, 7–9). Categorical data were summarised as n (%) and continuous data as median (interquartile range [IQR]: lower quartile-upper quartile). Chi-squared or exact permutation tests were used to test for association between categorical variables and frailty group. Jonckheere-Terpstra nonparametric tests were used to test for association between continuous variables and frailty group. Two-tailed tests with a significance level of 5% were used throughout. All analyses of this prospective longitudinal cohort study were exploratory, and no adjustment has been made for multiple comparisons.

The cumulative incidence of the composite endpoint’ first rehospitalisation and/or death during the first 12 months post-discharge was estimated using one minus the Kaplan-Meier survival function estimate. Plots of the cumulative incidence during the first 12 months post-discharge by age group, sex, CFS frailty group and Charlson score group were used to illustrate the relationships between these variables and log-rank tests used to test the univariable associations. Univariable Cox proportional hazard models of the time post-discharge to first rehospitalisation and/or death were used to estimate unadjusted hazard ratios (HR) and their 95% confidence intervals (CI). Adjusted HRs and 95% CIs were estimated using multivariable Cox models incorporating the variables of interest and potential confounders. The final model included frailty according to the CFS categories (mild-moderate frail vs. non-frail, and moderate-severe frailty vs. non-frail); sex (male sex vs. female sex); age separated into categories (76–80 years vs. ≤ 75 years; 81–85 years vs. ≤ 75 years; and ≥ 86 years vs. ≤ 75 years); and modified Charlson comorbidity score categories (score 1–2 vs. 0; and score ≥ 3 vs. 0).

## Results

Five hundred ninety-two participants had 12-month outcome data available and were included in the analyses. Baseline data were stratified by frailty status according to the Clinical Frailty Scale score, with a score of 1–4, classified as non-frail, a score of 5–6 classified as mild-moderately frail and 7–9 classified as severely frail [15]. The median age was 82 (IQR: 76–86) years, almost 60% of participants were female, and English was the primary language spoken. The majority of this cohort were frail, with 50% classified as mild-moderately frail and 21% classified as severely frail (see Table 1). Multimorbidity, polypharmacy, and poorer functional performance were associated with higher frailty. Follow-up data were available on all participants included in the analyses. If participants were unable to be contacted for telephone follow-up, then the follow-up data were extracted by the frailty clinical nurse specialist from the hospital’s electronic medical records, as per study protocol. Therefore, no participants ‘were lost to follow-up’. There was a statistically significant difference between the three frailty categories in terms of sex (female) (*p* = 0.005), age (p = < 0.001), body mass index (*p* = 0.039), AKPS score, (p = < 0.001), modified Charlson comorbidity score (*p* = 0.005), cognitive deficit and score (p = < 0.001 for both), albumin and haemoglobin level (p = < 0.001 for both) and total number of prescription medications (p = < 0.001). The prescription of specific classes of medications varied significantly across the frailty groups (see Table 1).

**Table 1:** Baseline characteristics stratified by CFS frailty group

	All *n* = 592	Non-frail (robust) CFS 1–4 *n* = 174 (29)	Mild- moderately frail CFS 5–6 *n* = 295 (50)	Severely frail CFS 7–9 *n* = 123 (21)	P*=
**Demographic characteristics**					
Sex – Female *n*,* (%)*	349 (59)	86 (49)	180 (61)	83 (66)	**0.005**
Age *median (IQR)*	82 (76–86)	78 (73–83)	82 (76–87)	85 (79–90)	**< 0.001**
BMI *median (IQR)*	27 (22–32)	27 (24–32)	27 (22–33)	25 (21–31)	**0.039**
Primary Language English *n*,* (%)*	484 (82)	144 (83)	245 (83)	94 (77)	0.345
Reside in Residential Aged Care Facility *n*,* (%)*	60 (10)	1 (0.6)	24 (8)	35 (29)	**< 0.001**
**Clinical characteristics**					
Australian Modified Karnofsky Performance Scale (pre-morbid)	60 (50–70)	80 (70–90)	60 (50–60)	40 (40–50)	**< 0.001**
Modified Charlson comorbidity Index	1 (0–2)	1 (0–2)	1 (0–2)	1 (0–3)	**0.005**
Assessment of Cognitive function completed *n*,* (%)*	433 (73)	134 (77)	211 (72)	88 (72)	0.391
Evidence of Cognitive deficit (according to a validated assessment tool) n, (%)	138 (23)	21 (12)	59 (20)	58 (47)	**<0.001**
4AT (Delirium and cognitive impairment screening) score (*n* = 398) *median (IQR)*	0 (0–3)	0 (0–1)	0 (0–2)	3 (0–4)	**< 0.001**
**Medication use**					
Antiepileptics *n*,* (%)*	36 (6)	8 (5)	21 (7)	7 (6)	0.533
Anxiolytics & hypnotics/benzodiazepines *n*,* (%)*	56 (10)	17 (10)	24 (8)	15 (12)	0.428
Analgesics *n*,* (%)*	443 (75)	116 (67)	231 (78)	96 (78)	**0.013**
Hypoglycaemics (oral, insulin) *n*,* (%)*	181 (32)	46 (26)	103 (35)	40 (33)	0.162
Antacids *n*,* (%)*	186 (31)	24 (13)	111 (38)	51 (42)	**< 0.001**
Loop Diuretics *n*,* (%)*	170 (29)	32 (19)	95 (32)	43 (35)	**0.001**
Antiarrhythmic (including Digoxin) *n*,* (%)*	191 (32)	63 (36)	98 (33)	30 (25)	0.088
Nitrate or other vasodilators *n*,* (%)*	81 (14)	19 (11)	47 (16)	15 (12)	0.270
Antidepressants *n*,* (%)*	123 (21)	26 (15)	66 (23)	31 (25)	0.063
Novel Oral Anticoagulants (NOACs) *n*,* (%)*	147 (25)	34 (20)	75 (26)	28 (40)	0.079
Warfarin *n*,* (%)*	41 (7)	11 (6)	20 (7)	10 (8)	0.825
Antihypertensive *n*,* (%)*	345 (58)	100 (56)	180 (61)	65 (53)	0.294
Proton-pump inhibitors *n*,* (%)*	131 (22)	52 (30)	61 (21)	18 (15)	**0.005**
Antiparkinsonian drugs *n*,* (%)*	20 (4)	9 (5)	11 (4)	4(3)	0.730
Statins *n*,* (%)*	351 (59)	111 (64)	179 (61)	61 (50)	**0.039**
Antiplatelet *n*,* (%)*	253 (43)	88 (51)	129 (44)	36 (29)	**0.001**
Antipsychotics *n*,* (%)*	39 (7)	6 (4)	19 (7)	14 (11)	**0.025**
Total number of prescription medications *n*,* (%)*	9 (7–13)	8 (6–11)	10 (7–14)	10 (7–14)	**< 0.001**
**Biochemistry**					
Creatinine	83 (66–111)	82 (66–99)	82 (66–112)	85 (65–127)	0.082
Albumin	31 (28–34)	33 (28–35)	31 (28–33)	29 (24–32)	**< 0.001**
Haemoglobin	120 (106–132)	124 (112–137)	120 (105–130)	113 (103–129	**< 0.001**

* Chi-squared or exact permutation test for categorical variables, Jonckheere-Terpstra nonparametric test for continuous variables

Legend: IQR; Inter quartile range; BMI; Body Mass Index; NOAC; Novel oral anticoagulant; 4AT – 4 A’s test: a score of 4 or more suggests delirium, a score of 1–3 suggests cognitive impairment

### 12-month outcomes

A total of 134 participants died (22.6%) within the 12-month follow-up period, comprising 29 (4.9%) participant deaths during baseline hospital admission and 105 (17.7%) participant deaths after hospital discharge. Of the 563 patients who were discharged from hospital, 316 (56.1%, 95% CI 52.0-60.2%) experienced at least one all-cause (non-elective, elective or emergency department visit) readmission and 73 (13.0%, 95% CI 10.4–16.0%) died without prior readmission within 12 months. Most of the 458 participants who were alive at 12 months were living at home (72%), a quarter were living in an aged care facility (25%), and the remainder were in hospital at the time of follow-up (3%).

### Cumulative incidence of composite rehospitalisation and/or death

**Fig. 1:**
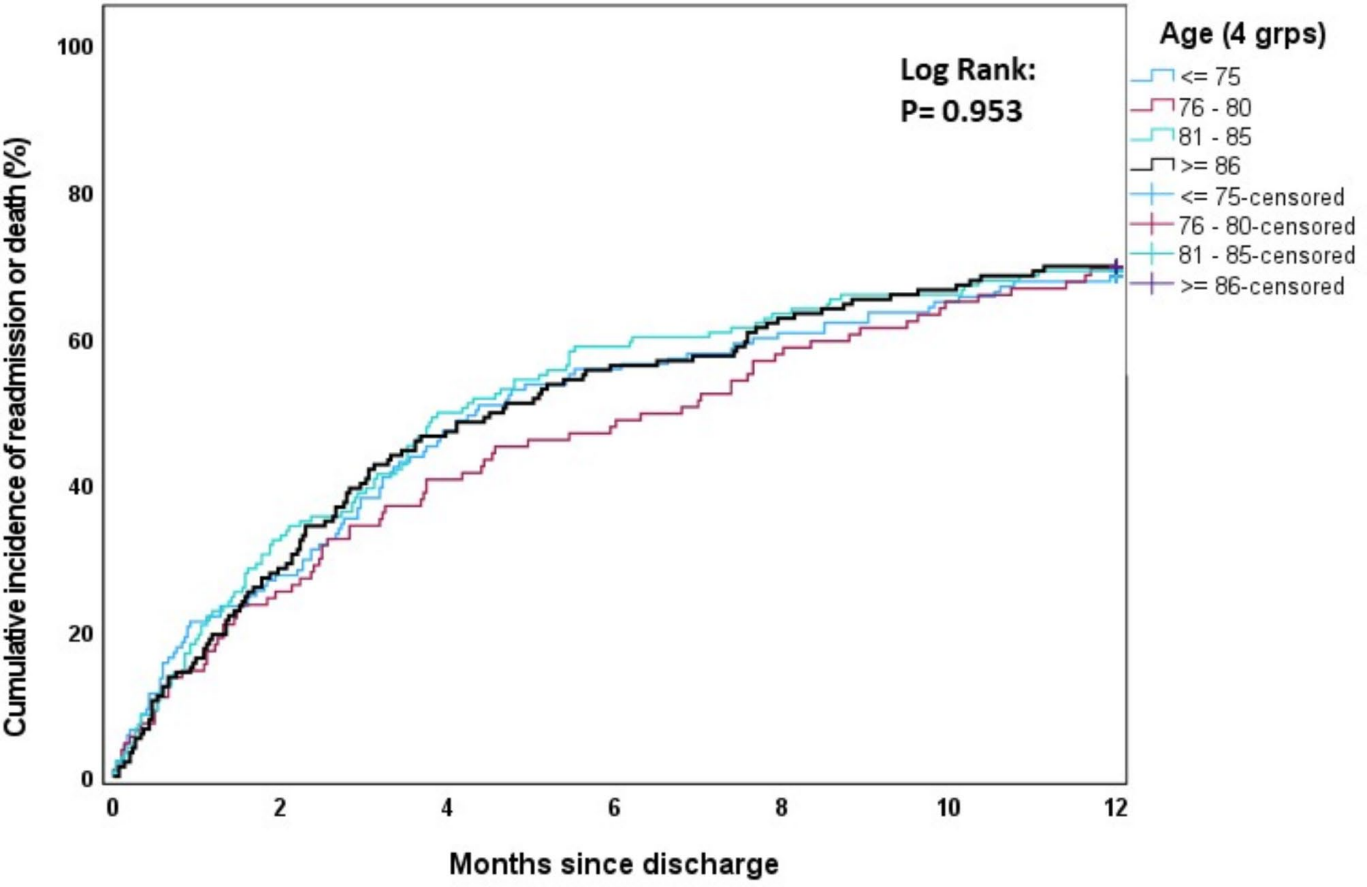
Cumulative incidence curves stratified by age group (≤ 75 years, 76–80 years, 81–85 years, ≥ 86 years)

**Fig. 2:**
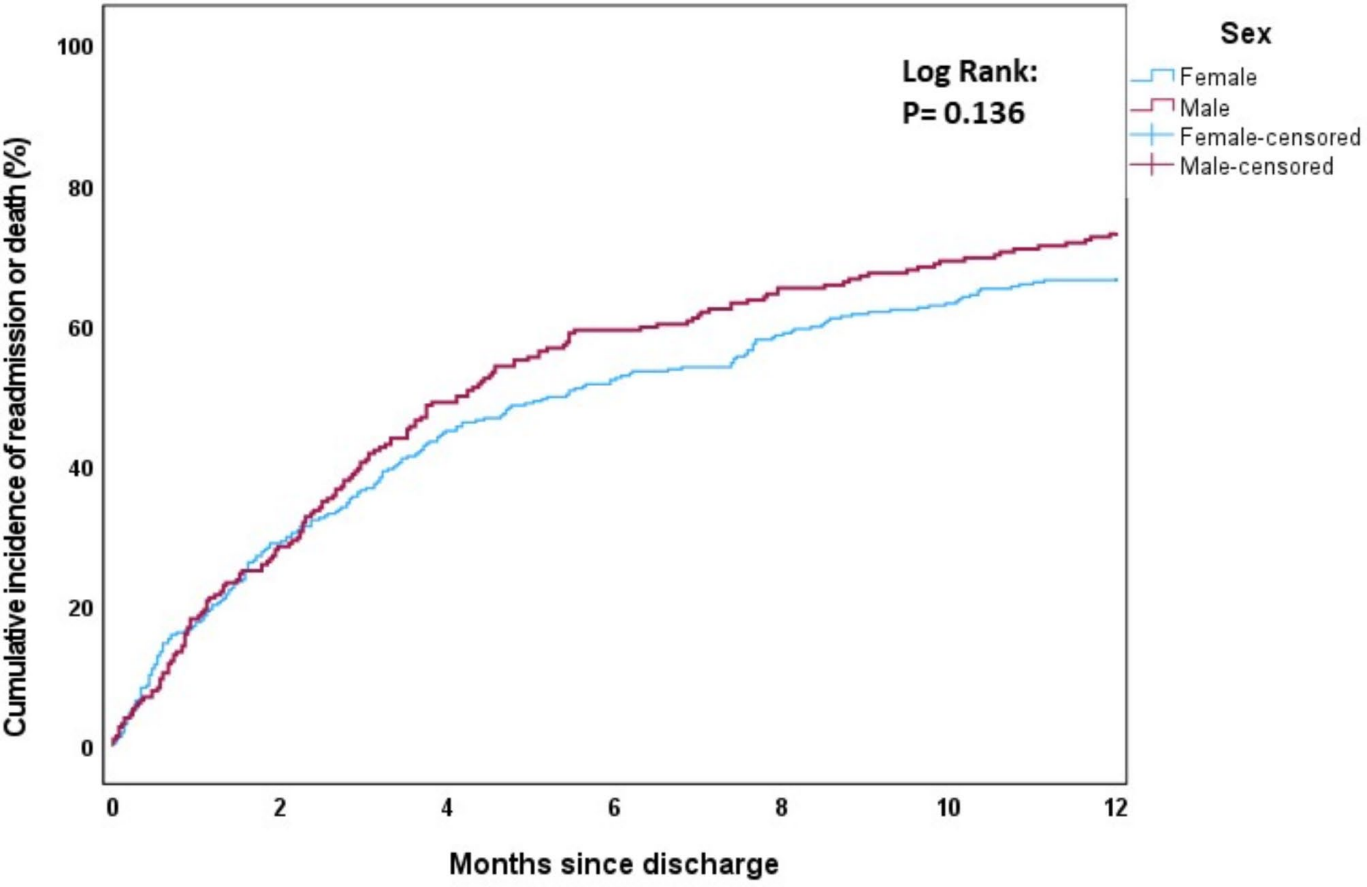
Cumulative incidence curves stratified by sex (Male, Female)

**Fig. 3:**
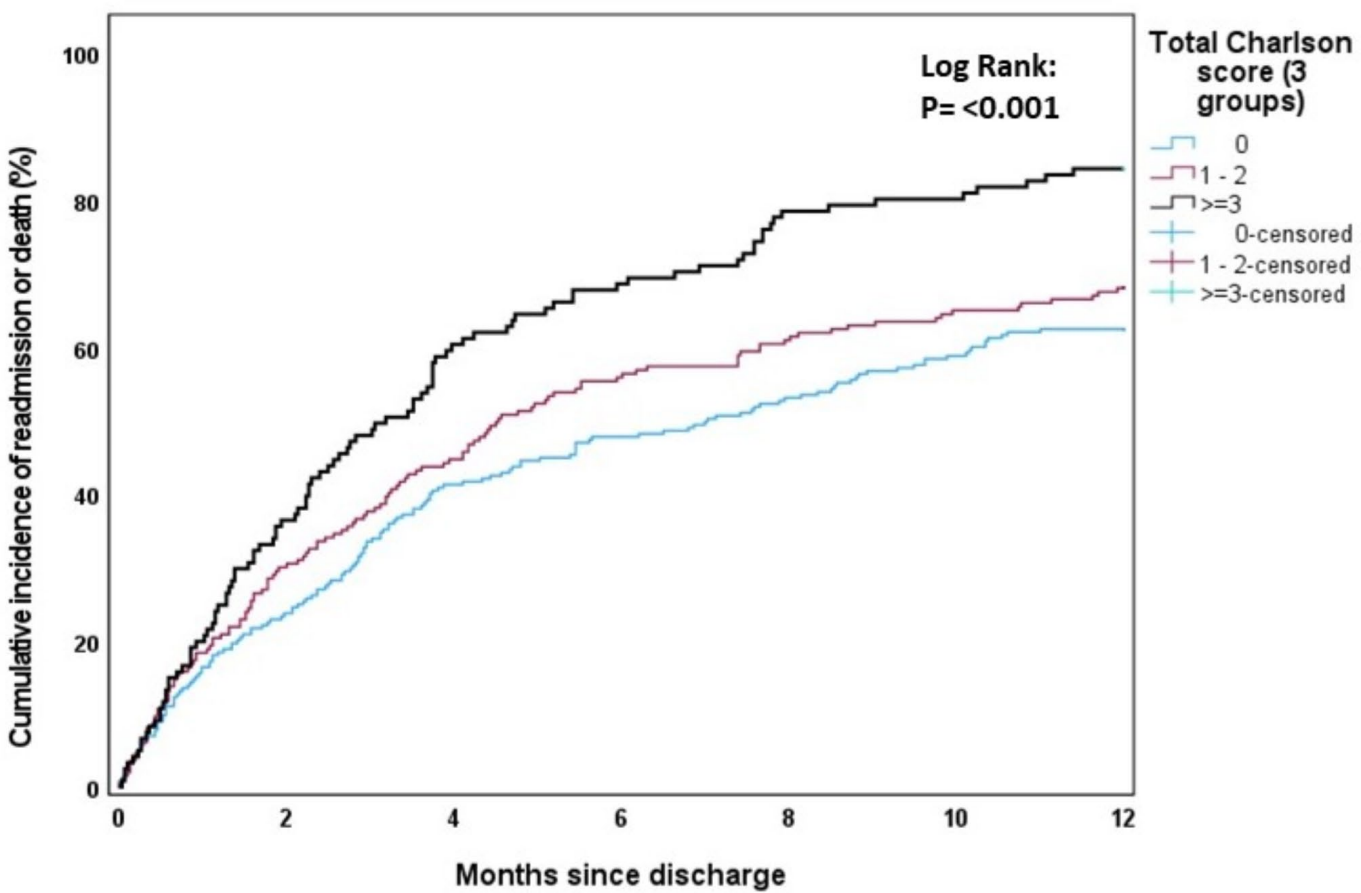
Cumulative incidence curves stratified by Charlson comorbidity score group (0, 1–2, ≥ 3)

**Fig. 4:**
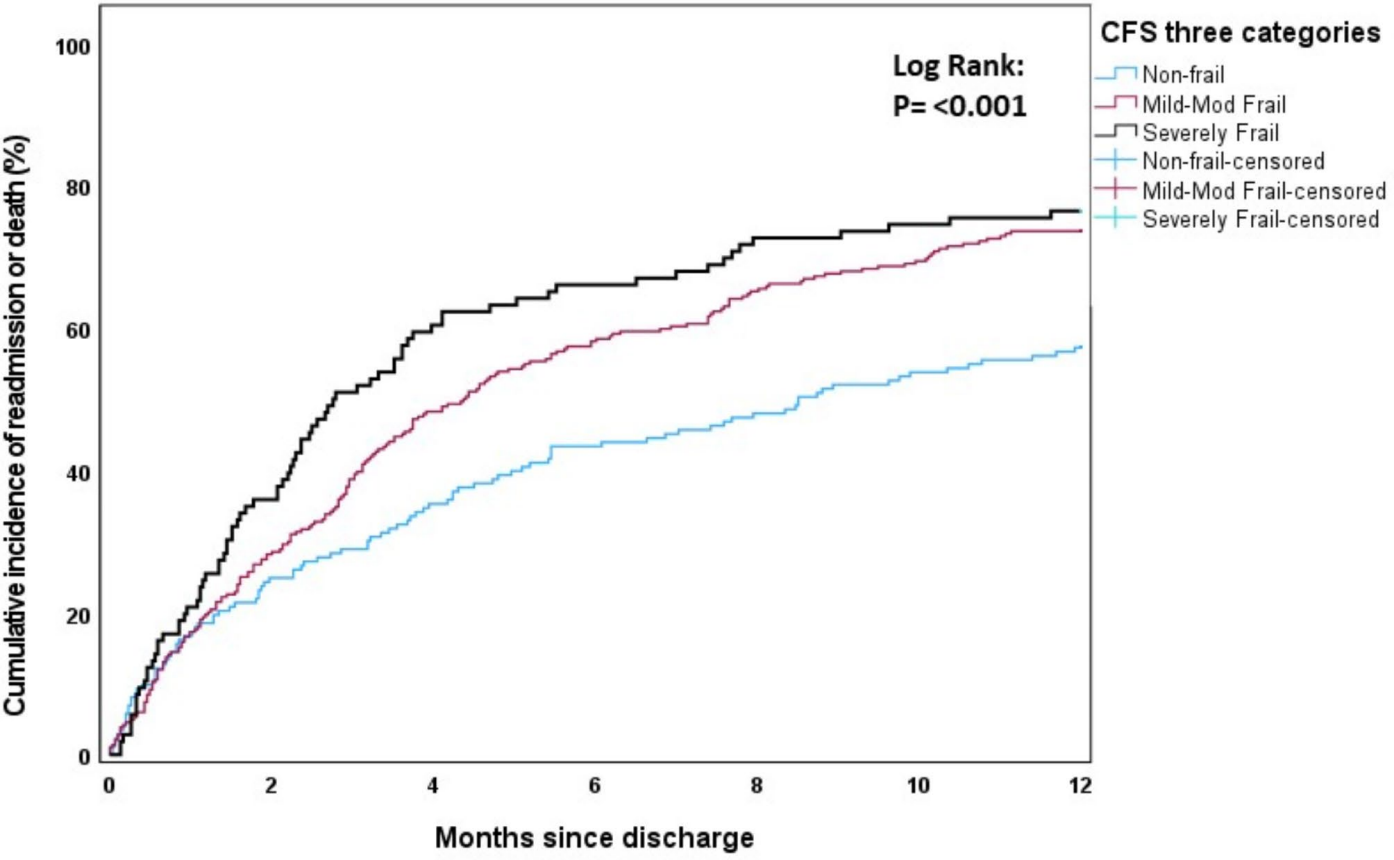
Cumulative incidence curves stratified by CFS group (non-frail, mild-moderately frail, severely frail)

The cumulative incidence of first rehospitalisation and/or death during the first 12 months post-discharge was estimated using one minus the Kaplan-Meier survival function estimate. The effects of age (Fig. 1), sex (Fig. 2), modified Charlson comorbidity score (Fig. 3) and frailty status (Fig. 4) on the cumulative incidence of first rehospitalisation and/or mortality during the first 12 months post-discharge are illustrated below. Increased cumulative incidence of first rehospitalisation and/or death during the first 12 months post-discharge was clearly associated with a higher Charlson comorbidity score and with a higher Frailty score (log-rank *p* < 0.001 for each variable).

### Predictors of rehospitalisation and/or mortality in the first 12 months post-discharge

The associated unadjusted hazard ratios (HR) and their 95% CIs estimated using univariable Cox proportional hazards models of the time post-discharge to first rehospitalisation and/or death are shown in Table 2 together with the adjusted HRs and 95% CIs from the multivariable Cox model incorporating all four variables. The independent predictors of the composite endpoint of rehospitalisation and/or death in the first 12 months post-discharge were the CFS frailty group, Charlson score group and sex. Compared to the ‘non-frail’ group (CFS 1–4), those who were severely frail (CFS 7–9) had an 85% increased risk of rehospitalisation and/or death (95% CI 1.36–2.52), and those who were mild-moderately frail (CFS 5–6) had a 52% increased risk after adjusting for effects of the other variables (95% CI 1.18–1.94). Compared to those with a Charlson score = 0, those with a Charlson score ≥ 3 had an adjusted 68% increased risk of rehospitalisation and/or death (95% CI 1.30–2.16). After adjusting for the effects of other variables, males had a 25% increased risk compared to females (95% CI 1.01–1.52).

**Table 2:** Unadjusted and adjusted hazard ratios (HR) with 95% CIs from Cox proportional hazards models of time from discharge to composite rehospitalisation and/or death in first 12 months*

Variable	unadjusted HR	95.0% CI for unadjusted HR		unadjusted *p*-value	adjusted HR	95.0% CI for adj HR		adjusted *p*-value
		Lower	Upper			Lower	Upper	
**Frailty: using the CFS (3 categories)**				**< 0.001**				**< 0.001**
**Mild-Mod vs. non-Frail**	1.50	1.18	1.91	**0.001**	1.52	1.18	1.94	**0.001**
**Severely Frail vs. non-Frail**	1.79	1.33	2.40	**< 0.001**	1.85	1.36	2.52	**< 0.001**
**Sex: Male vs. Female**	1.16	0.95	1.42	0.137	1.24	1.01	1.52	**0.043**
**Age in years (4 groups)**				*0.953*				*0.783*
**(76–80 years) vs. ≤ 75 years**	0.96	0.71	1.29	0.780	0.91	0.68	1.24	0.556
**(81–85 years) vs. ≤ 75 years**	1.04	0.79	1.37	0.788	0.91	0.69	1.21	0.512
**≥ 86 years vs. ≤ 75 years**	1.03	0.78	1.35	0.850	0.86	0.65	1.14	0.304
**Comorbidity: Total modified Charlson score** **(3 groups)**				***< 0.001***				***< 0.001***
**(Score of 1–2) vs. 0**	1.18	0.94	1.49	0.155	1.11	0.88	1.40	0.383
**Score ≥ 3 vs. 0**	1.78	1.38	2.28	**< 0.001**	1.68	1.30	2.16	**< 0.001**

* The final variables included in the adjusted multivariate model were frailty, sex, age, and modified Charlson comorbidity index score

Legend: CFS: clinical frailty score

## Discussion

This prospective analysis of 592 adults enrolled into the Western Sydney Clinical Frailty registry demonstrates that pre-morbid frailty assessed using the simple, pictorial CFS is a powerful independent predictor of rehospitalisation and/or mortality within 12 months post-discharge. A recent systematic review examining the association between CFS and adverse health outcomes in older adults in acute care settings supports our findings, reporting that all studies (*n* = 29) demonstrated that a CFS score could independently predict multiple adverse outcomes, such as rehospitalisation, mortality, length-of-stay, functional decline [17]. Another recent study evaluating the CFS in adults ≥ 70 years admitted to geriatric acute care reported that the CFS showed high inter-rater reliability between consultant doctors, nurses and other medical officers (intraclass correlation coefficient = 0.859, 95% CI: 0.827-0.885, *P* < 0.001) [21]. Furthermore, the CFS can be rapidly completed, which is helpful in a busy clinical setting.

A higher Charlson comorbidity score (≥ 3) was also an independent predictor of the composite endpoint. Higher frailty scores were significantly associated with higher Charlson comorbidity index scores. Frailty and comorbidity are closely related, with both occurring due to aging-related processes, yet they are distinct, with frailty reflecting physiological vulnerability and comorbidities reflecting specific disease burden [22]. These results highlight the importance of including an assessment of comorbidity alongside a frailty assessment. Including frailty and comorbidity measures could provide a more comprehensive understanding of overall health status, allowing for more accurate risk stratification and personalised care planning [23].

Polypharmacy was significantly associated with higher frailty scores in this cohort of older adults admitted to acute geriatric services. People classified as frail were significantly more likely to be prescribed analgesics, antacids, loop diuretics, and antipsychotics. However, they were significantly less likely to be prescribed a statin, antiplatelet or proton pump inhibitor. Our results provide real-world information regarding the use of medicines in older, frail populations. Currently, there is a lack of guidelines to inform prescribing (and deprescribing) for frail older adults and limited evidence about the pharmacokinetics and pharmacodynamics in the context of frailty [24]. The International Union of Basic and Clinical Pharmacology Geriatric Committee recently recommended that frailty be assessed in clinical trials involving older adults, both at baseline and as an outcome for efficacy and safety [25]. Therefore, it is important to routinely assess frailty in the clinical setting and clinical trials to inform future practice on the quality use of medicines.

Our results suggest that routine frailty screening using the CFS for older adults presenting to acute geriatric settings is not only feasible but clinically useful. For example, frailty scores could help inform shared decision-making conversations on the likelihood of readmission and/or death after discharge. These data could also improve decision-making in the context of invasive procedures (e.g. endoscopy), de-prescribing, and advanced care planning at a local level in Western Sydney and more broadly at a national level. There is future hope for real-time frailty assessment via Electronic Medical Records and dashboards [26–28], which would eliminate costly human resources needed to undertake more time-consuming performance-based frailty measures.

### Clinical implications and future directions

Recently, the HARMONY model (acHieving dAta-dRiven quality iMprovement to enhance frailty Outcomes using a learNing health sYstem), a new frailty learning health system model of implementation science and practice improvement, was applied to the Western Sydney Frailty Clinical Frailty Registry [29]. Clinicians at the study site were presented with interim results from the frailty registry, and in general, there was surprise at the high mortality rate in those with severe frailty. This has important implications for acute geriatric medicine care. On the one hand, this could represent missed opportunities for preventative care but may also support the idea that these patients are in the final years of their life. In such cases, the patient’s preferences for goals of care should be discussed, including advanced care planning. It also demonstrates the importance of routinely collecting post-discharge outcome data that might be important in managing future patients.

The Western Sydney Clinical Frailty Registry recently had ethical amendments approved for ‘opt-out’ consent procedures. A consumer advisory group with Aged Care and Rehabilitation services consumers revealed that consumers believed the post-discharge phone calls provided within the registry follow-up should be standard practice. Having opt-out consent is less burdensome for participants, researchers, and clinicians. It also allows greater access to the increased follow-up post-discharge for all people admitted to the Aged Care and Rehabilitation services at Blacktown Mount Druitt Hospitals. Further, this consent model aims to reduce study selection and recruitment bias.

As per a recent recommendation by the Australian Registry of Clinical Quality Registries, we have also been approved to collect additional patient-reported outcome measures (PROMS) on willing and able participants. The PROMs include quality of life, self-reported frailty, and depression and will be collected at baseline and repeated at the 12-month follow-up.

Readmission was common in this cohort, with one in two patients rehospitalised within 12 months. The research team is conducting qualitative research with consumers, clinicians, and expert stakeholders to explore hospital transition for older adults with frailty. Consumer priorities were brain health and functional independence. Further, the use of the hospital was often viewed as an entitlement of older Australians, contrasting hospital management and policy priorities of reducing readmissions and emergency department presentations. We intend to co-design and pilot an intervention to improve the hospital transition experience.

Strengths and Limitations.

The oldest and most frail patients are often left out of clinical trials. We have demonstrated the feasibility of prospectively recruiting and following up a large cohort of frail older people from a busy acute geriatric medicine service. As noted, follow-up data was available on all participants because this was permitted from the participant’s electronic medical record. This ongoing study presents an opportunity for trials within a cohort study design to evaluate frailty interventions and data linkage studies. Our consumer advisory panel supported the research and helped revise our registry procedures.

The major weakness of our registry was the inevitable selection bias; for example, the eligibility criteria for the Western Sydney Clinical Frailty Registry stipulates that participants must speak English or have a family member who can provide consent on their behalf, which has inevitably resulted in a selected population. We also did not have the resources to recruit all admitted patients. However, we have assembled a large cohort of older adults with differing levels of frailty that has allowed us to explore the effects of frailty on important outcomes. As mentioned previously if participants were unable to be contacted for follow-up, information regarding rehospitalisation and death were extracted from medical records or publicly available death registries. While every effort was made to avoid reporting errors, using two different data collection methods may have resulted in discrepancies. Finally, as the frailty registry utilises routinely collected data, it is possible that misclassification bias and underreporting may have impacted data quality. These limitations may have affected our results and reduced the generalisability of our findings.

## Conclusions

Frailty is very common in older adults admitted to acute geriatric services, and it has significant implications for adverse outcomes, treatment and health service planning. Assessing frailty using the CFS is feasible and is independently predictive of rehospitalisation and mortality. Our findings suggest that integrating frailty assessment into clinical practice goes beyond simple risk stratification, offering valuable clinical insights for tailored management strategies.

## Data Availability

The datasets used and/or analysed during the current study are available from the corresponding author upon reasonable request.
